# Inflammatory proteins mediate male erectile dysfunction via plasma metabolites

**DOI:** 10.1093/sexmed/qfae027

**Published:** 2024-05-31

**Authors:** Zhen Kang, Zhuo-Rui Zhang, Zhi-Yuan Feng, Long-Shen Dong, Junfeng Yang

**Affiliations:** State Key Laboratory of Primate Biomedical Research, Institute of Primate Translational Medicine, Kunming University of Science and Technology, Kunming, China; Department of Urology, The First People’s Hospital of Yunnan Province, The Affiliated Hospital of Kunming University of Science and Technology, Kunming, China; State Key Laboratory of Primate Biomedical Research, Institute of Primate Translational Medicine, Kunming University of Science and Technology, Kunming, China; Department of Urology, The First People’s Hospital of Yunnan Province, The Affiliated Hospital of Kunming University of Science and Technology, Kunming, China; State Key Laboratory of Primate Biomedical Research, Institute of Primate Translational Medicine, Kunming University of Science and Technology, Kunming, China; Department of Urology, The First People’s Hospital of Yunnan Province, The Affiliated Hospital of Kunming University of Science and Technology, Kunming, China; State Key Laboratory of Primate Biomedical Research, Institute of Primate Translational Medicine, Kunming University of Science and Technology, Kunming, China; Department of Urology, The First People’s Hospital of Yunnan Province, The Affiliated Hospital of Kunming University of Science and Technology, Kunming, China; State Key Laboratory of Primate Biomedical Research, Institute of Primate Translational Medicine, Kunming University of Science and Technology, Kunming, China; Department of Urology, The First People’s Hospital of Yunnan Province, The Affiliated Hospital of Kunming University of Science and Technology, Kunming, China

**Keywords:** erectile dysfunction, inflammatory proteins, plasma metabolites, Mendelian randomization

## Abstract

**Background:**

There are no clear conclusions as to whether inflammatory proteins and plasma metabolites influence erectile dysfunction (ED).

**Aim:**

In this research, we used Mendelian randomization (MR) analysis to discover a causal relationship between inflammatory proteins, plasma metabolites, and ED.

**Methods:**

Raw data with ED, inflammatory proteins, and plasma metabolites were obtained from the MRC IEU OpenGWAS and FinnGen database. After a series of screenings, the remaining single nucleotide polymorphisms were selected as instrumental variables or MR analysis to assess the relationship between genetically predicted inflammatory proteins or plasma metabolites and the pathogenesis of ED.

**Outcomes:**

The relationship between inflammatory factors and ED was fully analyzed and elaborated.

**Results:**

In the inverse variance–weighted method, there exists a significant causal relationship between 4 types of genetically predicted inflammatory proteins and 50 types of plasma metabolites with the incidence of ED. The primary discovery is that 3 inflammatory proteins, fibroblast growth factor 5, interleukin-22 receptor subunit alpha-1, and protein S100-A12, can impact the risk of ED through plasma metabolites.

**Clinical Implications:**

ED metabolites and inflammatory proteins are also closely associated with cardiovascular diseases, warranting further exploration.

**Strengths and Limitations:**

Our analysis is based on a European population, limiting its generalizability, the genome-wide association study dataset for ED has a relatively small number of cases, and we hope for larger genome-wide association study datasets for future validation.

**Conclusion:**

This study has identified that inflammatory proteins can influence ED through plasma metabolites.

## Introduction

Erectile dysfunction (ED) is a complex physiological process involving neural and vascular events. The global prevalence of ED varies significantly, ranging from 14% to 48%, with the highest rates observed in the United States and Southeast Asia, as opposed to Europe.^1^ More than affecting psychosocial well-being, ED is increasingly recognized as a precursor to cardiovascular diseases, including myocardial infarction and cerebrovascular events.^2^ Therefore, ED should be seen not only as a matter affecting quality of life, but also as a critical indicator of potential cardiovascular issues.^3^

Recent research employing Mendelian randomization (MR) analysis of existing data has revealed previously underrecognized risk factors for ED, including insomnia,^4^ depression,^5^ and periodontal disease.^6^ These findings invite further investigation into the role of inflammation in the development of ED. Inflammation’s dysregulated responses are known to contribute to tissue damage and play a central role in the onset of various diseases. Exploring this area could shed new light on the mechanisms behind ED and offer fresh insights into its understanding.^7^

Free radicals produced during inflammation can damage tissues, impairing the normal functioning of penile tissues^8^; chronic diseases like diabetes and hypertension, which are associated with inflammation, may have long-term detrimental effects on the nervous system, subsequently affecting erectile function.^9^^,^^10^ Research has pinpointed certain inflammatory proteins (IPs), such as tumor necrosis factor α (TNF-α) and interleukin (IL)-6, as potential mediators of inflammation’s adverse impact on erectile function through their role in regulating cellular signaling pathways.^11^ These findings highlight a link between inflammation and erectile function and suggest avenues for future research to explore.

Plasma metabolites are small molecular compounds found in the plasma that mirror the body’s metabolic state, encompassing amino acids, lipids, sugars, nucleotides, and other small molecules. Certain plasma metabolites, such as dihydrotestosterone, phosphodiesterase, and prostaglandin E1, play crucial roles in maintaining erectile function and are primary targets for ED treatment. Additionally, substances like L-arginine, vascular endothelial growth factor, and brain-derived neurotrophic factor^12^ have been explored for their potential effects on ED, possibly by influencing the cavernous nerve. Previous studies exploring the association between ED and IPs or metabolites mostly used traditional clinical study designs, and most of them were observational studies, which had problems such as small sample sizes, uncontrollable confounding factors, and inability to randomly assign diseases. MR analysis is a novel approach for exploring causal relationships between diseases. Genetic variants such as single nucleotide polymorphisms (SNPs) were used as instrumental variables, and random assignment during gamete formation was used to simulate the random assignment process of disease. In addition, the sample size of genome-wide association study (GWAS) data based on this method is generally large, which can be used for bidirectional causal association analysis. This study aimed to explore the causal relationship between IPs or metabolites in ED by MR analysis.

## Methods

### Study population

The research population was primarily derived from 3 distinct projects. The ED cohort originated from ED-FinnGen (https://storage.googleapis.com/finngen-public-data-r9/summary_stats/finngen_R9_ERECTILE_DYSFUNCTION.gz), the most recent ED-related dataset as of 2021, comprising a total of 166 309 participants (2205 cases and 164 104 controls). The IPs-related GWAS dataset was analyzed by Zhao et al^13^ using the Olink Target platform across 11 cohorts (14 824 individuals of European descent), yielding genomic protein quantitative trait loci for 91 IPs. The plasma metabolome dataset was sourced from the Canadian Longitudinal Study on Aging cohort, encompassing 8299 participants. Chen et al^14^ identified GWAS data associated with 1091 plasma metabolites and 309 metabolite ratios relevant to human disease progression.

### Study design

**Figure 1:**
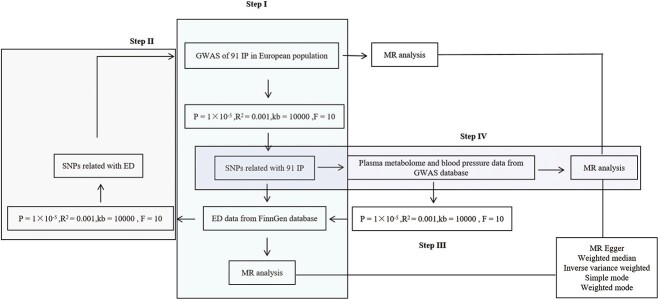
Illustration of the associations in each step of this study. In step I, 91 IPs act as exposure variables, with ED as as the outcome variable, to screen for IPs associated with ED. In step II, ED serves as the exposure variable, utilizing the identified IP from step I as outcomes to screen for IPs exhibiting a reverse causal relationship with ED. Only those IPs lacking a reverse causal relationship are suitable for subsequent mediation MR analysis. In step III, 1400 metabolites act as exposure variables, and ED serves as the outcome, to identify metabolites associated with ED. In step IV, using the IPs identified in step II as exposure variables and the metabolites identified in step III as outcomes, IPs associated with metabolites are screened. These IPs potentially influence the occurrence of ED by modulating specific metabolite levels. It is crucial to note that qualified instrumental variables must not be duplicated, so instrumental variables used in step III need to exclude those already used in step I.

This study employed 2-sample MR, reverse MR, and mediation MR to investigate the potential causal relationship between IP levels and ED, as well as whether plasma metabolites could serve as intermediate factors concurrently associated with both (Figure 1).

### Instrumental variable selection criteria

To establish a robust MR model, the selected SNPs as instrumental variables must satisfy 3 assumptions: (1) relevance (the chosen SNPs must be associated with the exposure of interest to a certain degree); (2) independence (the SNPs are not associated with any confounders involved in the exposure-outcome pathway); and (3) exclusivity (the SNPs affect the study outcome solely through their impact on the exposure, meaning that the SNPs do not have significant pleiotropy). To meet the relevance criterion and obtain the required number of SNPs for analysis, included SNPs should have genome-wide significance (*P* < 1 × 10^−5^). To ensure the independence of SNPs, those with an r^2^ <0.001 are selected, and each pair of SNPs must be more than 10 000 kb apart to avoid linkage disequilibrium due to physical proximity. The exclusivity of SNPs is evaluated using the intercept term in MR-Egger regression, and SNPs with significant pleiotropy are excluded.

## Pleiotropy and heterogeneity

The research assessed heterogeneity and pleiotropy between SNPs using Cochran’s Q statistic and MR-Egger (Supplementary Table 1).

### Statistical methods

The primary statistical analysis for MR was conducted using the TwoSampleMR package in R 4.1.2 (R Foundation for Statistical Computing). Five methods, MR Egger, weighted median, inverse variance weighting (IVW), simple mode, and weighted mode, were employed to test the causal relationship between inflammatory factor SNPs and ED. The IVW method assumes that all SNPs in MR analysis are valid, combining the Wald ratios of each SNP into an overall weighted effect, and the results obtained from IVW are considered the primary findings. The RMediation package was utilized to calculate the 95% confidence interval (CI) of the mediation effect.

## Results

### The association between IPs and ED

**Figure 2:**
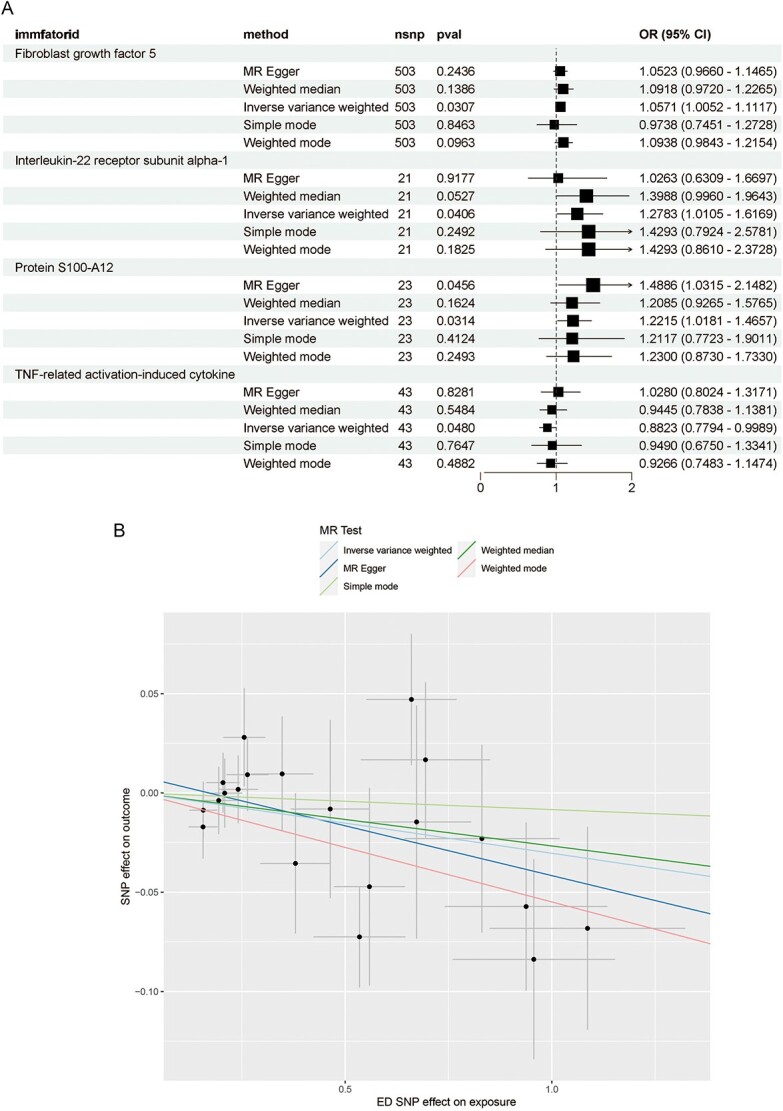
(A) Forest plot to visualize the causal effects of inflammatory proteins with erectile dysfunction (ED). (B) Scatter plots of genetic associations with ED against the genetic associations with tumor necrosis factor-related activation-induced cytokine level. The slopes of each line represent the causal association for each method. From top to bottom, the first line says simple mode method estimates, said weighted median estimate in the second line, third line according to the inverse variance weighted estimate, the fourth line according to Mendelian randomization – egger estimates, article 5 of the line shows the weighted mode estimates.

A total of 91 IPs were identified based on instrumental variable selection criteria, with varying quantities of SNPs per IP ranging from 20 to 500 (https://github.com/uro66/inflammatory-proteins-SNPs). Five methods were employed to estimate the causal relationship between IPs and ED. Our primary focus was on the IVW analysis, revealing that 3 IPs—fibroblast growth factor 5 (FGF5), IL-22 receptor subunit alpha-1 (IL22RA1), and protein S100-A12—increased the risk of ED, while TNF-related activation-induced cytokine (TRANCE) decreased the risk (Figure 2A). In reverse MR analysis, using ED SNPs as the exposure factor, we found an inverse causal relationship between IP TRANCE and ED, with an odds ratio (OR) of 0.970 (95% CI, 0.942 to 0.998; *P* = .040) using the IVW method (Figure 2B), while the other 3 IPs showed no reverse causal connections with ED.

### The plasma metabolome as a mediating factor

**Figure 3:**
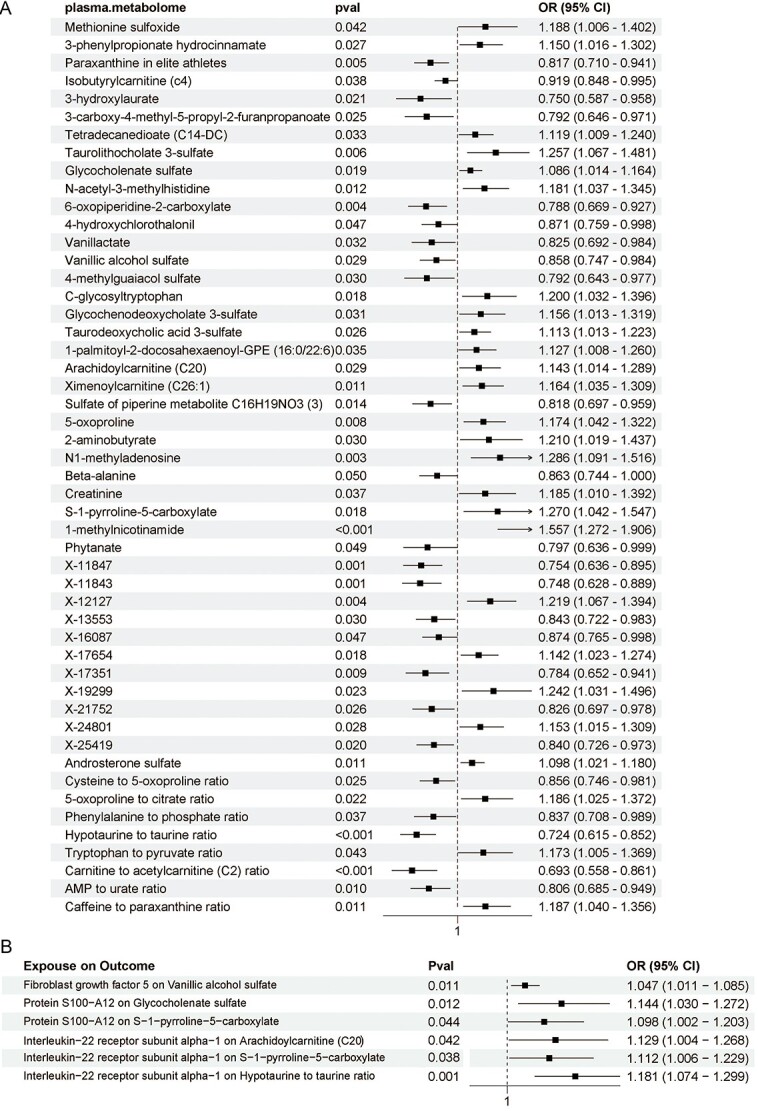
(A) Forest plot to visualize the causal effects of the plasma metabolome with erectile dysfunction (ED) (inverse variance–weighted [IVW] results). (B) Forest plot to visualize the causal effects of inflammatory proteins with the plasma metabolome (IVW results).

Based on instrumental variable selection criteria, 1400 SNPs of plasma metabolome were obtained, and the SNPs can be obtained at https://github.com/uro66/plasma-metabolom. Using 5 methods revealed that 50 plasma metabolites were associated with the risk of ED (*P* < .05). Our main focus was on the IVW results, indicating that the carnitine-to-acetylcarnitine (C2) ratio was the most likely biomarker to reduce the risk of ED (OR, 0.69; 95% CI, 0.56 to 0.86), while 1-methylnicotinamide was a metabolite most likely to increase the risk of ED (OR, 1.56; 95% CI, 1.27 to 1.91). Detailed results can be found in Figure 3A. Using the SNPs of 3 IPs—FGF5, IL22RA1, and protein S100-A12—as exposure factors and 50 plasma metabolomes as outcome factors, MR analysis revealed that these proteins significantly elevated the levels of 5 plasma metabolites, which can serve as intermediate factors between IPs and ED. Detailed results are presented in Figure 3B.

### IPs influence ED through the plasma metabolome

The analysis of 5 plasma metabolites as the mediators from IPs to ED revealed intriguing interactions. FGF5 was found to elevate the levels of the metabolite vanillic alcohol sulfate, which in turn reduced the risk of ED. However, the overall effect of FGF5 increased the risk of ED, suggesting the presence of other mediators counteracting the slowing effect of vanillic alcohol sulfate on ED. Protein S100-A12, another IP, was associated with an increase in the levels of metabolites S-1-pyrroline-5-carboxylate and glycocholenate sulfate, both of which raised the risk of ED. IL22RA1 increased the levels of vanillic alcohol sulfate, arachidoylcarnitine (C20), and the hypotaurine-to-taurine ratio, with the former two also increasing the risk of ED. Notably, the hypotaurine-to-taurine ratio, a metabolic indicator, decreased the risk of ED (mediation β = −0.054; 95% CI, −0.107 to −0.001) (Table 1).

**Table 1:** Details of IPs and plasma metabolites MR analysis data used in this study.

Exposure	Mediator	Outcome	Total effect	Direct effect A	Direct effect B	Mediation effect (β)	Mediated proportion (%)
Fibroblast growth factor 5	Vanillic alcohol sulfate	ED	0.056 (0.005 to 0.106)	0.046 (0.011 to 0.082)	−0.154 (−0.291 to −0.016)	−0.007	—
Protein S100-A12	S-1-pyrroline-5-carboxylate		0.200 (0.017 to 0.382)	0.094 (0.002 to 0.185)	0.239 (0.041 to 0.436)	0.022	11.188
	Glycocholenate sulfate		0.200 (0.017 to 0.382)	0.135 (0.030 to 0.240)	0.083 (0.014 to 0.152)	0.011	5.571
Interleukin-22 receptor subunit alpha-1	Glycocholenate sulfate		0.245 (0.010 to 0.480)	0.106 (0.006 to 0.206)	0.083 (0.014 to 0.152)	0.009	3.574
	Arachidoylcarnitine (C20)		0.245 (0.010 to 0.480)	0.121 (0.004 to 0.237)	0.134 (0.014 to 0.254)	0.016	6.613
	Hypotaurine-to-taurine ratio		0.245 (0.010 to 0.480)	0.166 (0.071 to 0.262)	−0.323 (−0.485 to −0.161)	−0.054	—

Values are β (95% confidence interval), unless otherwise indicated. The total effect indicates the effect of relative inflammatory proteins on ED, direct effect A indicates the effect of inflammatory proteins on the plasma metabolome, direct effect B indicates the effect of the plasma metabolome on ED, and the mediation effect indicates the effect of inflammatory proteins on ED through the plasma metabolome. Total effect, direct effect A, and direct effect B were derived by inverse variance weighting, and the mediation effect was obtained by multiplying the β values of the direct effect.

Abbreviation: ED, erectile dysfunction.

## Discussion

ED results from systemic vascular occlusive diseases and is closely associated with endothelial dysfunction. IPs have been well documented to promote endothelial cell proliferation, migration, and angiogenesis, ultimately playing a role in vasculogenesis. In this study, we newly identified 4 IPs that alter the likelihood of ED development. Furthermore, as the most basic and abundant components in plasma, metabolites have also been discovered to potentially act as intermediary molecules transmitting the pathogenic effects of IPs.

First, TRANCE regulates immune responses and bone remodeling. In murine models, it can induce angiogenesis through metabolites such as Src and phospholipase C. Our findings suggest that TRANCE may reduce the risk of ED, likely due to its role in promoting angiogenesis. Second, ED can be an early manifestation of coronary and peripheral vascular diseases.^15^ Elevated levels of the calcium-binding protein S100-A12 are associated with reduced cardiac output and increased risk of heart failure, also serving as an independent risk factor for atherosclerosis.^16^ The intermediary molecule, taurocholic acid sulfate, linked to S100-A12, also correlates with an increased risk of atrial fibrillation, further confirming the close relationship between ED and cardiovascular diseases. Pyrroline-5-carboxylate reductase (P5CRs) acts as another intermediary between S100-A12 and ED, primarily catalyzing proline biosynthesis. Elevated levels of proline may affect the structure and function of vascular walls, increase oxidative stress, and impair vasodilation. Third, in mice with myocardial hypertrophy, IL22RA1 levels increase in parallel with markers of myocardial hypertrophy.^17^ IL-22 is also an important marker for heart failure. Our study found that IL22RA1 increases the ratio of taurine to hypotaurine, substances abundant in male reproductive organs. The antihypertensive effects of taurine have been reported, in which it can reduce blood pressure in male rats with hypertension by downregulating endothelial nitric oxide synthase in the aortic arch^18^ and enhancing testosterone levels and nitric oxide production, thus improving sexual response and mating ability in male rats.^19^ Although IL22RA1 increases the risk of ED, it also boosts taurine production, a seemingly paradoxical outcome. We hypothesize that IL22RA1 mediates some currently unknown risk factors that offset its promotive effects on taurine. For instance, our study discovered that IL22RA1 could also increase levels of taurocholic acid sulfate, which, when bound to free bile acids, participates in fat digestion and absorption as bile salts, consuming some taurine. However, direct evidence for this is lacking and further experiments are needed.

Regarding ED risk factors, numerous MR studies have focused on previously overlooked causes, including insomnia, snoring, obesity, and depression.^20^ However, the most extensively researched cause remains cardiovascular disease, providing a foundational approach for this study to investigate overlooked IPs using MR, ultimately uncovering previously undervalued factors.

This study has several limitations. First, our analysis is based on a European population, limiting its generalizability. Second, there is a relative scarcity of GWAS datasets for ED, and we hope for larger datasets in the future for validation. Third, despite measures taken to identify and eliminate outlier variants, we cannot exclude the possibility of pleiotropy affecting our results. Last, the impact of our intermediary factors on the outcomes is relatively low, necessitating further research to quantify the influence of other mediators.

## Conclusion

First, TRANCE can decrease the risk of ED. Second, the IP S100-A12 can increase the risk of ED by elevating the levels of S-1-pyrroline-5-carboxylate and glycocholenate sulfate. Last, IL22RA1 increases the levels of vanillic alcohol sulfate, arachidoylcarnitine (C20), and the hypotaurine-to-taurine ratio. While vanillic alcohol sulfate and arachidoylcarnitine (C20) increase the risk of ED, the hypotaurine-to-taurine ratio decreases the risk of ED.

## Supplementary Material

supplementary_table_1_qfae027

## Data Availability

The datasets presented in this study can be found in online repositories.
